# *Amphiroa fragilissima* as a bioactive resource: exploring its antioxidant, anti-biofilm, anti-inflammatory, and antibacterial potential for dental applications

**DOI:** 10.2340/biid.v12.45099

**Published:** 2025-12-11

**Authors:** Dileepkumar Hemamalini, S. Shantha Sundari, K.M. Shahul Hameed Faizee, Sivakamavalli Jeyachandran

**Affiliations:** a Department of Orthodontics and Dentofacial Orthopedics, Saveetha Dental College, Saveetha Institute of Medical and Technical Science (SIMATS), Saveetha University, Chennai, India; b Department of Orthodontics and Dentofacial Orthopedics, Sathayabama Dental College and Hospital, Sathyabama Institute of Science and Technology, Sathyabama University, Chennai, India; c Lab in Biotechnology and Biosignal Transduction, Department of Orthodontics, Saveetha Dental College and Hospital, Saveetha Institute of Medical and Technical Sciences (SIMATS), Saveetha University, Chennai, India

**Keywords:** Amphiroa Fragilisima, Bioactive polysaccharide, white spot lesion

## Abstract

**Aim and objectives:**

To evaluate the antibacterial, antibiofilm, antioxidant, and anti-inflammatory properties of *Amphiroa fragilissima* and assess its potential for dental and orthodontic use.

**Materials and methods:**

Methanolic extracts of *A. fragilissima*, collected from Rameshwaram, India, were tested against *Streptococcus mutans, Enterococcus faecalis, Escherichia coli*, and *Shigella sonnei* using the Kirby-Bauer method. Antibiofilm activity was analyzed via Crystal Violet staining. Antioxidant potential was assessed using 2,2-Diphenyl-1-picrylhydrazyl radical scavenging, and anti-inflammatory activity was measured via a bovine serum albumin assay.

**Results:**

The extract showed dose-dependent antibacterial activity, with maximum inhibition observed at 100 µg/mL. Biofilm inhibition also increased with concentration. Antioxidant assays revealed significant radical scavenging activity, with results comparable to controls at higher concentrations. Anti-inflammatory testing showed reduced protein denaturation in treated samples, with effects similar to the positive control and significantly better than the blank.

**Conclusion:**

*Amphiroa fragilissima* demonstrates strong antibacterial, antibiofilm, antioxidant, and anti-inflammatory activities, along with remineralization potential due to its calcium-rich composition. These properties support its potential as a natural, multifunctional agent for dental and orthodontic applications. Further *in vivo* studies are recommended to validate its clinical use.

## Introduction

*Amphiroa fragilissima* is a species of marine algae, commonly known as a red algae. It belongs to the family *Amphiaroaceae* and is typically found in marine environments, particularly in the intertidal zones. This species is notable for its delicate and fragile appearance, which is reflected in its name, ‘*fragilissima*’, which comes from the Latin word for fragile [1].

It is a member of the red algae group (Rhodophyta), which is characterized by pigments such as phycoerythrin, giving them their reddish color. Like many red algae, *A. fragilissima* can play an important role in the ecosystem by contributing to the structure of marine habitats, providing food, and supporting biodiversity [1].

The high mineral content and excellent biocompatibility of calcareous red algae such as *A. fragilissima* position them as promising candidates for applications in bone grafts and dental tissue repair. *Amphiroa* species, characterized by their rich deposits of calcium carbonate (mainly as high-magnesium calcite and aragonite), offer a natural source of minerals essential for hard tissue regeneration. Their composition suggests significant potential in promoting bone turnover and supporting the regeneration of bone and dental structures, particularly in the context of dental implants and periodontal therapies [2, 3].

In addition to their mineral richness, *Amphiroa* species contain bioactive molecules with antibacterial, anti-inflammatory, and antioxidant properties. Studies have reported that extracts from *A. fragilissima* demonstrate significant antimicrobial activity against oral pathogens such as *Streptococcus mutans* and *Lactobacillus spp.*, which are primary contributors to dental caries and biofilm formation [4, 5].

The antioxidant activity of *A. fragilissima* has been attributed to its phenolic and sulfated polysaccharide content, which can scavenge free radicals and reduce oxidative stress – a key factor in inflammatory oral diseases. Anti-inflammatory effects have also been observed, with studies showing a downregulation of pro-inflammatory mediators when cells are treated with *Amphiroa*-derived compounds [5, 6].

The dissolution and demineralization of dental hard tissues such as enamel, dentin, and cementum are inevitable processes driven by environmental and genetic factors. Enamel, being the most mineralized and externally exposed tissue, is highly susceptible to demineralization. Partial enamel dissolution plays a role in the dynamic balance of mineral content – primarily calcium and phosphate ions – within oral fluids, maintaining a homeostatic state [7, 8].

Traditionally, fluoride-based therapies have been the cornerstone of efforts to regulate this mineral equilibrium and prevent dental caries. However, growing concerns over the systemic exposure to fluoride have led many regions, particularly in the European Union, the United States, and Canada, to reassess community water fluoridation policies [8, 9].

This contentious debate highlights the necessity of developing safe, sustainable, and effective alternatives to fluoride. *A. fragilissima* with its natural mineralization capacity and bioactive profile, offers a compelling, biocompatible substitute that could aid in enamel remineralization, reduce microbial colonization, and promote overall oral health.

This study aims to investigate the potential of calcareous red macroalgae *A. fragilissima* for dental applications, specifically focusing on its antioxidant, anti-inflammatory, antibiofilm, and antibacterial properties. Owing to its high calcium content and previously reported nutraceutical benefits related to bone health and mineral metabolism, *A. fragilissima* may offer a novel, sustainable strategy for enhancing enamel remineralization and oral health maintenance.

## Materials and methods

### Sample collection and preparation

**Figure 1:**
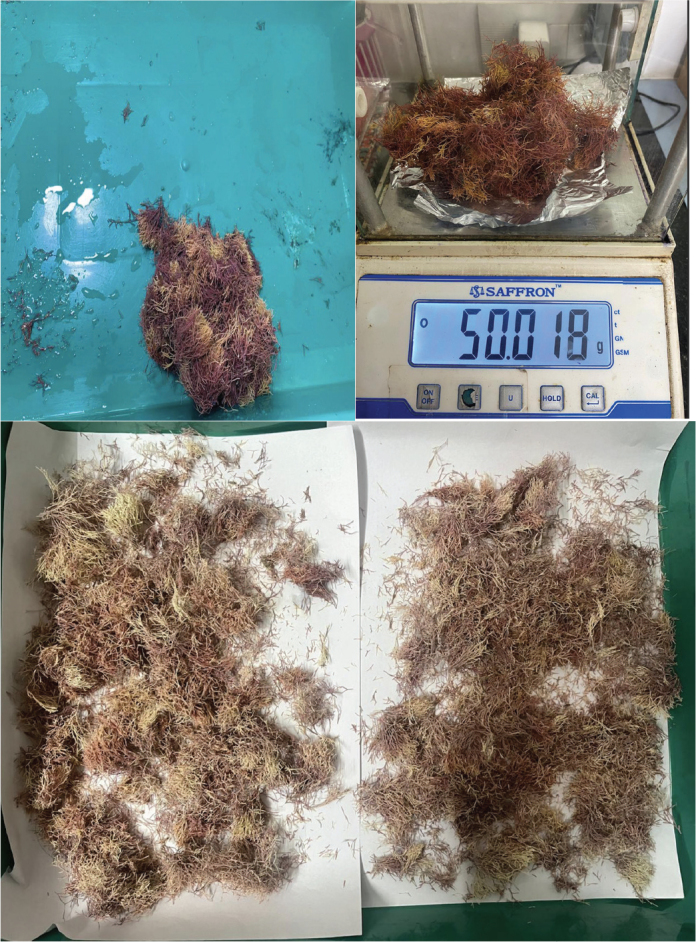
Processing, drying and fine powdering of *Amphiroa fragilisima*.

The sample, which was collected from several locations in Rameshwaram, Tamil Nadu, India was determined to be *A. fragilissima*. The sample was cleaned in seawater to remove any debris (Figure 1) and allowed to dry for 2 days in the sun. It was then rinsed with tap water to remove any remaining sand and salt and allowed to dry in the sun for 7 days before being finely chopped with a scalpel to create a powder.

### Solvent extract

The *A. fragilissima* powder (40 g) was combined with three volumes of methanol, following the method outlined by Terada et al. [10, 11]. The mixture was homogenized at 10,000 × g for 2 minutes using a homogenizer. Afterward, the homogenate was stirred continuously at room temperature for 30 minutes. The mixture was then centrifuged at 5,000 × g for 10 minutes at room temperature in a cooling centrifuge (REMI C-24 Plus, Goregaon [East], Mumbai, Maharashtra, India) to remove any undissolved debris.

### Antibacterial activity

The antibacterial efficacy of the *A. fragilisima* extract was evaluated using the Kirby-Bauer method [12]. The bacteria selected for testing the antibacterial activity included *S. mutans* (Sdc_ortho5 16S Ribosomal RNA gene, partial sequence GenBank: OQ947767.1), *Enterococcus faecalis* (NCT34 16S Ribosomal RNA gene, partial sequence GenBank: OM283553.1), *Shigella sonnei* (NCT34 16S Ribosomal RNA gene, partial sequence GenBank: OM283552.1), and *Escherichia coli* (16S Ribosomal RNA gene, partial sequence, GenBank: U00096.3). The bacterial inoculum was cultured overnight in nutrient broth and a fixed volume was added to 10 mL aliquots of nutrient agar, mixed, and then poured over a nutrient agar base in sterile Petri dishes, forming the bacterial lawn. The surfaces of the plates were then inoculated with 200 μL of bacterial suspension. Using the well diffusion method, wells containing 25, 50, 75, and 100 μL of *A. fragilisima* extract were placed onto the agar plates. Each Petri dish was sealed individually to prevent contamination and media evaporation. The plates were incubated at 37°C for 24–48 hours. Antibacterial effects were indicated by the clear zone of inhibition surrounding the wells, and the diameter of each inhibition zone was measured in millimeters using a Vernier caliper (Baker Gauges India Pvt. Ltd. – Pune, Maharashtra).

### Biofilm inhibition assay

Biofilm production and development were evaluated using light microscopy after Crystal Violet staining, following the protocol outlined by Zhou [13]. Biofilms were cultured in the wells of a 24-well microtiter plate and stained with Crystal Violet stain (Hi-media, India). Freshly cultured bacterial suspensions of *S. mutans*, *E. coli*, *E. faecalis*, and *S. sonnei* were added to each well of a 24-well polystyrene microtiter plate (Corning, Mumbai, India), which had previously been lined with sterile glass cover slips for microscopic examination. The plates were incubated from 48 to 72 hours, and the cover slips were then collected. Glass squares measuring 1 × 1 cm were used to assess the inhibitory effects of *A. fragilisima* extracts on *in vitro* biofilm formation. Biofilms were allowed to form on these glass squares, which were placed in 24-well polystyrene plates containing *A. fragilisima* extracts (25–100 µg/mL) and incubated for 24 hours at 30°C. A sterile medium without bacteria served as the control. After incubation, the glass squares were retrieved and rinsed twice with Phosphate Buffered Solution (PBS) before microscopic examination. The biofilms were stained with 6% Crystal Violet, and qualitative analysis was performed using an Olympus CX21i LED Microscope (Olympus Corporation, Tokyo, Japan) at 40x magnification.

### DPPH radical scavenging activity

The DPPH (2,2-Diphenyl-1-picrylhydrazyl) radical scavenging activity was assessed following the method described by Shekar et al. [14]. To prepare the extract, 10 mg of *A. fraglissima* was dissolved in 10 mL of methanol. A stock solution of DPPH was prepared by dissolving 24 mg of DPPH (Sisco Research Laboratories Pvt. Ltd. [SRL], India) in 100 mL of methanol. This stock solution was stored under refrigeration until further use. Before analysis, it was diluted with methanol to obtain a working solution with an absorbance of approximately 0.98 ± 0.02 at 517 nm. For the assay, 3 mL of the DPPH working solution was mixed with 100 μL of either the standard solution (ascorbic acid as the positive control) or the *A. fraglissima* extract in a glass vial. The absorbance was then measured at 517 nm using a Shimadzu UV-1900i Spectrophotometer (Shimadzu Corporation, Kyoto, Japan) over a period of 30 minutes. PBS served as the negative control (blank). The percentage of radical scavenging activity was calculated for extract concentrations of 10, 20, 30, 40, and 50 µg/mL. The DPPH concentration at time t after reaction with the antioxidant sample is represented as DPPH] T = t. The standard utilized was ascorbic acid.

*% Radical Scavenging Activity (RSA) = (control-sample/control)*100

### Bovine serum albumin assay

The anti-inflammatory potential of *A. fraglissima* was evaluated using a modified version of the bovine serum albumin (BSA) assay as described by Williams et al. [15]. A 0.4% (w/v) BSA solution was prepared using Tris-buffered saline by dissolving one tablet in 15 mL of deionized water, resulting in 0.05 M Tris and 0.15 M sodium chloride at pH 7.6 (25°C). The pH of this solution was then adjusted to 6.4 using diluted glacial acetic acid. Stock solutions of *A. fraglissima* were prepared in methanol at a concentration of 50 μg/mL (0.005%, w/v). From this stock, aliquots of 5.0, 10, and 20 µL – corresponding to final concentrations of 0.25, 0.50, and 1.00 µg/mL – were added to test tubes containing 1 mL of 0.4% BSA solution (prepared by dissolving 1 g of BSA in 80 mL of PBS, then adjusting the final volume to 100 mL with distilled water).

Both negative (dimethyl sulfoxide, DMSO) and positive (aspirin) controls were prepared and treated similarly. All tubes were heated in a water bath at 72°C for 10 minutes, then cooled to room temperature for 20 minutes. Turbidity, indicating the degree of protein denaturation, was measured at 660 nm using a Shimadzu UV-1900i Spectrophotometer (Shimadzu Corporation, Tokyo, Japan), with distilled water used as the blank. Each experiment was conducted in duplicate, and mean absorbance values were recorded. The percentage inhibition of protein denaturation – an indicator of anti-inflammatory activity – was calculated relative to the negative control using the following formula:

*%Inhibition = Abs of standard – Abs of sample/Abs of standard*100

All assays were conducted in triplicate using three independent biological replicates, each comprising separately prepared extracts. Within each biological replicate, technical triplicates were performed, and the mean ± SD was calculated from biological replicate means.

### Statistical analysis

The results were analyzed by one way analysis of variance (ANOVA) and in case of significant differences by post hoc test (Tukey’s Honest Significant Difference [HSD]). The statistical analyses were performed using Statistical Package for the Social Sciences (SPSS) version 25 and the level of significance was set at *p* = 0.05.

## Results

### Antibacterial activity

**Figure 2:**
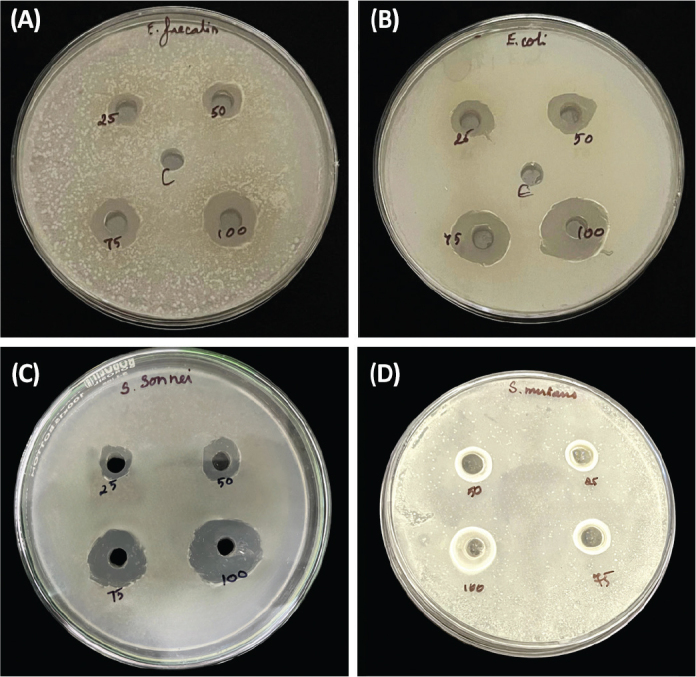
Antibacterial activity of *Amphiroa fragilissima* against (A) *Enterococcus faecalis* (B) *Escherichia coli* (C) *Streptococcus mutans* and (D) *Shigella sonnei*.

**Figure 3:**
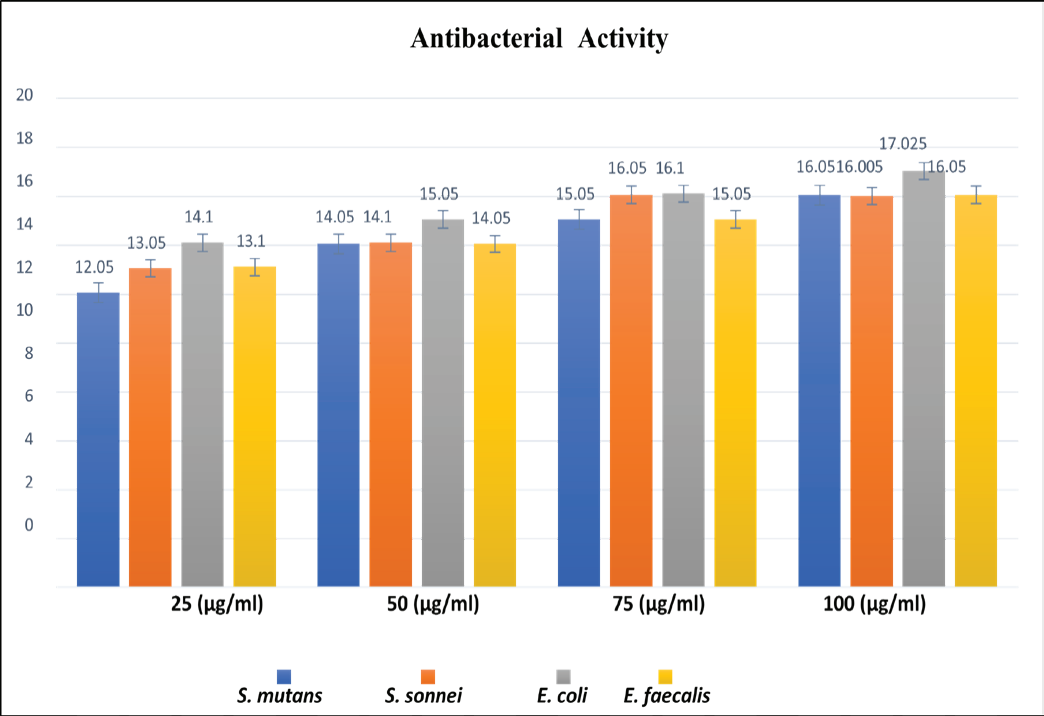
Antibacterial activity (Zone of inhibition) of *Amphiroa Fragilisima* at varied concentration.

The antibacterial activity of the tested samples was evaluated against *E. faecalis, E. coli, S. sonnei, and S. mutans* at different concentrations of *A. fraglissima* (25, 50, 75, and 100 µg/mL). The mean inhibition zone diameters (mm) and standard deviations as represented in Tables 1 and 2 and Figures 2 and 3. The results demonstrated a clear dose-dependent increase in the zone of inhibition for all tested organisms, indicating enhanced efficacy at higher concentrations.

**Table 1:** Antibacterial response (diameter of inhibition zone) of four different concentration of *Amphiroa fragilisima* against the four bacterial groups.

Concentration (µg/mL)	Groups	Mean	SD	*P*
**@25**	***S. mutans***	12.0500	0.07071	< 0.001
	***S. sonnei***	13.0500	0.07071	
	***E. coli***	14.1000	0.14142	
	***E. faecalis***	13.1000	0.14142	
**@50**	***S. mutans***	14.0500	0.07071	< 0.001
	***S. sonnei***	14.1000	0.00000	
	***E. coli***	15.0500	0.07071	
	***E. faecalis***	14.0500	0.07071	
**@75**	***S. mutans***	15.0500	0.07071	< 0.001
	***S. sonnei***	16.0500	0.07071	
	***E. coli***	16.1000	0.14142	
	***E. faecalis***	15.0500	0.07071	
**@100**	***S. mutans***	16.0500	0.07071	< 0.001
	***S. sonnei***	16.0050	0.00707	
	***E. coli***	17.0250	0.03536	
	***E. faecalis***	16.0500	0.07071	

**Table 2:** Post hoc analysis of antibacterial responses between the four bacterial groups at various concentration of *Amphiroa fragilisima*.

Concentration (µg/mL)	Group comparing	Group compared	Mean difference	Std. error	*P*
**@25**	***S. mutans***	***S. sonnei***	-1.00000*	0.11180	0.003
		***E. coli***	-2.05000*	0.11180	< 0.001
		***E. faecalis***	-1.05000*	0.11180	0.002
	***S. sonnei***	***S. mutans***	1.00000*	0.11180	0.003
		***E. coli***	-1.05000*	0.11180	0.002
		***E. faecalis***	-0.05000	0.11180	0.967
	***E. coli***	***S. mutans***	2.05000*	0.11180	< 0.001
		***S. sonnei***	1.05000*	0.11180	0.002
		***E. faecalis***	1.00000*	0.11180	0.003
	***E. faecalis***	***S. mutans***	1.05000*	0.11180	0.002
		***S. sonnei***	0.05000	0.11180	0.967
		***E. coli***	-1.00000*	0.11180	0.003
**@50**	***S. mutans***	***S. sonnei***	-0.05000	0.06124	0.845
		***E. coli***	-1.00000*	0.06124	< 0.001
		***E. faecalis***	0.00000	0.06124	1.000
	***S. sonnei***	***S. mutans***	0.05000	0.06124	0.845
		***E. coli***	-0.95000*	0.06124	< 0.001
		***E. faecalis***	0.05000	0.06124	0.845
	***E. coli***	***S. mutans***	1.00000*	0.06124	< 0.001
		***S. sonnei***	0.95000*	0.06124	< 0.001
		***E. faecalis***	1.00000*	0.06124	< 0.001
	***E. faecalis***	***S. mutans***	0.00000	0.06124	1.000
		***S. sonnei***	-0.05000	0.06124	0.845
		***E. coli***	-1.00000*	0.06124	< 0.001
**@75**	***S. mutans***	***S. sonnei***	-1.00000*	0.09354	0.002
		***E. coli***	-1.05000*	0.09354	0.001
		***E.faecalis***	0.00000	0.09354	1.000
	***S. sonnei***	***S.mutans***	1.00000*	0.09354	0.002
		***E.coli***	-0.05000	0.09354	0.946
		***E. faecalis***	1.00000*	0.09354	0.002
	***E. coli***	***S. mutans***	1.05000*	0.09354	0.001
		***S. sonnei***	0.05000	0.09354	0.946
		***E. faecalis***	1.05000*	0.09354	0.001
	***E. faecalis***	***S. mutans***	0.00000	0.09354	1.000
		***S. sonnei***	-1.00000*	0.09354	0.002
		***E. coli***	-1.05000*	0.09354	0.001
**@100**	***S. mutans***	***S. sonnei***	0.04500	0.05315	0.831
		***E. coli***	-0.97500*	0.05315	< 0.001
		***E. faecalis***	0.00000	0.05315	1.000
	***S. sonnei***	***S. mutans***	-0.04500	0.05315	0.831
		***E. coli***	-1.02000*	0.05315	< 0.001
		***E. faecalis***	-0.04500	0.05315	0.831
	***E. coli***	***S. mutans***	0.97500*	0.05315	< 0.001
		***S. sonnei***	1.02000*	0.05315	< 0.001
		***E. faecalis***	0.97500*	0.05315	< 0.001
	***E. faecalis***	***S. mutans***	0.00000	0.05315	1.000
		***S. sonnei***	0.04500	0.05315	0.831
		***E. coli***	-0.97500*	0.05315	< 0.001

At 25 µg, the mean inhibition zones ranged from 12.05 mm (*S. mutans*) to 14.10 mm (*E. coli*), while at 100 µg, they increased substantially, with *E. coli* showing the highest susceptibility (17.03 ± 0.035 mm), followed by *S. mutans* and *E. faecalis* (16.05 ± 0.07 mm) and *S. sonnei* (16.00 ± 0.007 mm). The overall differences in microbial inhibition across concentrations were statistically significant (*p* < 0.001).

Post hoc analysis (Tukey’s multiple comparisons) revealed that at 25 µg, *E. coli* exhibited significantly greater inhibition compared to *S. mutans* (mean difference: 2.05 mm, *p* < 0.001), *S. sonnei* (1.05 mm, *p* = 0.002), and *E. faecalis* (1.00 mm, *p* = 0.003). At 50 µg, *E. coli* continued to show significantly higher inhibition than *S. mutans* and *S. sonnei* (*p* < 0.001), while no significant differences were observed between *S. mutans*, *S. sonnei*, and *E. faecalis*.

At 75 µg and 100 µg, *E. coli* remained the most sensitive organism with statistically significant differences compared to all other species (*p* < 0.001). Notably, at the highest concentration (100 µg), the inhibition zones among *S. mutans*, *S. sonnei*, and *E. faecalis* were not significantly different from each other (*p* > 0.8), suggesting a saturation of antimicrobial response at this level.

### Antibiofilm activity

**Figure 4:**
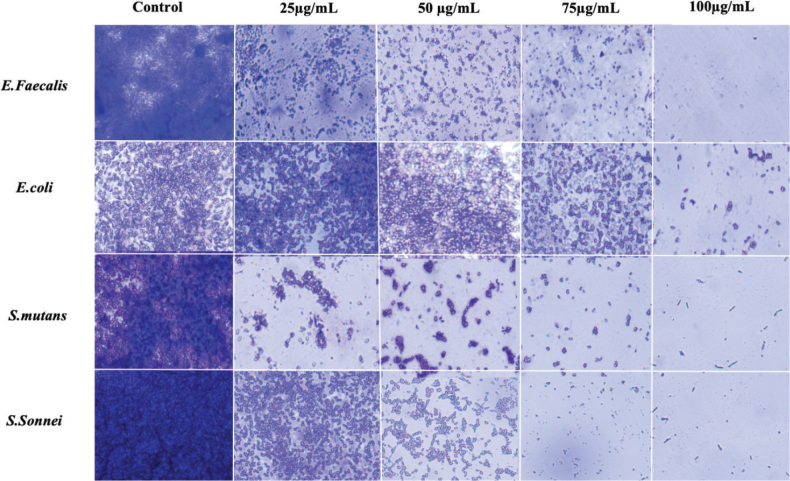
Light microscopic images showing antibiofilm activity at different concentrations of *Amphiroa fragilisima* against (A) *Enterococcus faecalis* (B) *Escherichia coli* (C) *Streptococcus mutans* (D) *Shigella sonnei*.

The antibiofilm activity of *A. fragilissima* extract against *S. mutans*, *E. coli*, and *E. faecalis* demonstrated a clear concentration-dependent enhancement, with measurable inhibition observed from the lowest tested concentration of 25 µg/mL and a progressive, substantial increase in biofilm suppression at 50 µg/mL, 75 µg/mL, and reaching maximal efficacy at 100 µg/mL, as depicted in Figure 4.

### Antioxidant activity

**Figure 5:**
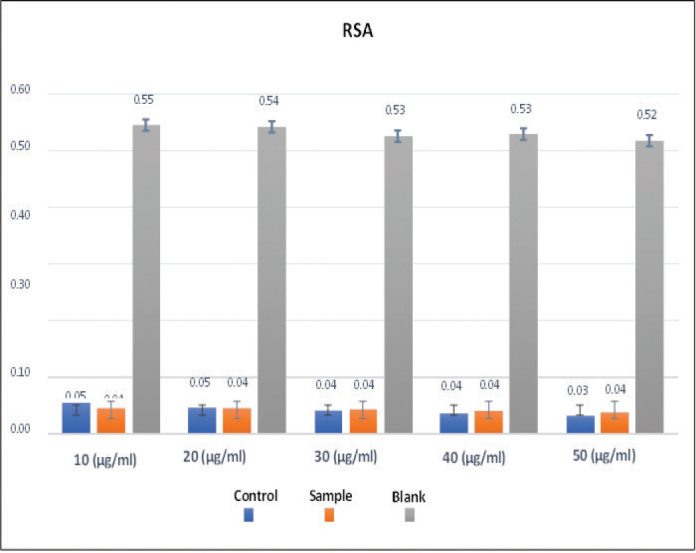
Antioxidant activity (% radical scavenging activity) of *Amphiroa Fragilisima* at varied concentration.

The antioxidant response of *A. fragilissima* was evaluated at five different concentrations (10, 20, 30, 40, and 50 µg/mL) and compared to a blank and control group (Table 3 and Figure 5). At each concentration, there were significant differences among the blank, control, and *A. fragilissima* groups (*p* < 0.001). Although no significant differences were observed between the control and *A. fragilissima* groups, post hoc analysis showed that the absorbance values of the blank were consistently and significantly higher than those of both the control and sample groups (Table 4), indicating that both the control and *A. fragilissima* treatments exhibited notable radical scavenging activity. However, *A. fragilissima* did not significantly outperform the control.

**Table 3:** Antioxidant response (measured in nm) for five different concentration of *Amphiroa fragilissima*.

Concentration (µg/mL)	Group	Mean	Std.deviation	*P*
**@10**	**Control**	0.0545	0.00150	< 0.001
	**Sample**	0.0445	0.00450	
	**Blank**	0.5450	0.01100	
**@20**	**Control**	0.0455	0.00050	< 0.001
	**Sample**	0.0445	0.00250	
	**Blank**	0.5420	0.00200	
**@30**	**Control**	0.0405	0.00050	< 0.001
	**Sample**	0.0425	0.00750	
	**Blank**	0.5255	0.00250	
**@40**	**Control**	0.0355	0.00050	< 0.001
	**Sample**	0.0400	0.00900	
	**Blank**	0.5290	0.00400	
**@50**	**Control**	0.0315	0.00050	< 0.001
	**Sample**	0.0375	0.00750	
	**Blank**	0.5175	0.00350	

**Table 4:** Post hoc analysis on antioxidant responses between three groups over different concentrations of *Amphiroa fragilissima*.

Concent ration	Group comparing	Group compared	Mean difference	Std. error	*P*
**@10**	**Control**	**Sample**	0.01000	0.00565	0.257
		**Blank**	–0.49050*	0.00565	< 0.001
	**Sample**	**Control**	–0.01000	0.00565	0.257
		**Blank**	–0.50050*	0.00565	< 0.001
	**Blank**	**Control**	0.49050*	0.00565	< 0.001
		**Sample**	0.50050*	0.00565	< 0.001
**@20**	**Control**	**Sample**	0.00100	0.00153	0.797
		**Blank**	–0.49650*	0.00153	< 0.001
	**Sample**	**Control**	–0.00100	0.00153	0.797
		**Blank**	–0.49750*	0.00153	< 0.001
	**Blank**	**Control**	0.49650*	0.00153	< 0.001
		**Sample**	0.49750*	0.00153	< 0.001
**@30**	**Control**	**Sample**	–0.00200	0.00373	0.857
		**Blank**	–0.48500*	0.00373	< 0.001
	**Sample**	**Control**	0.00200	0.00373	0.857
		**Blank**	–0.48300*	0.00373	< 0.001
	**Blank**	**Control**	0.48500*	0.00373	< 0.001
		**Sample**	0.48300*	0.00373	< 0.001
**@40**	**Control**	**Sample**	-0.00450	0.00465	0.621
		**Blank**	-0.49350*	0.00465	< 0.001
	**Sample**	**Control**	0.00450	0.00465	0.621
		**Blank**	-0.48900*	0.00465	< 0.001
	**Blank**	**Control**	0.49350*	0.00465	< 0.001
		**Sample**	0.48900*	0.00465	< 0.001
**@50**	**Control**	**Sample**	-0.00600	0.00391	0.341
		**Blank**	-0.48600*	0.00391	< 0.001
	**Sample**	**Control**	0.00600	0.00391	0.341
		**Blank**	-0.48000*	0.00391	< 0.001
	**Blank**	**Control**	0.48600*	0.00391	< 0.001
		**Sample**	0.48000*	0.00391	< 0.001

### Anti-inflammatory

**Figure 6:**
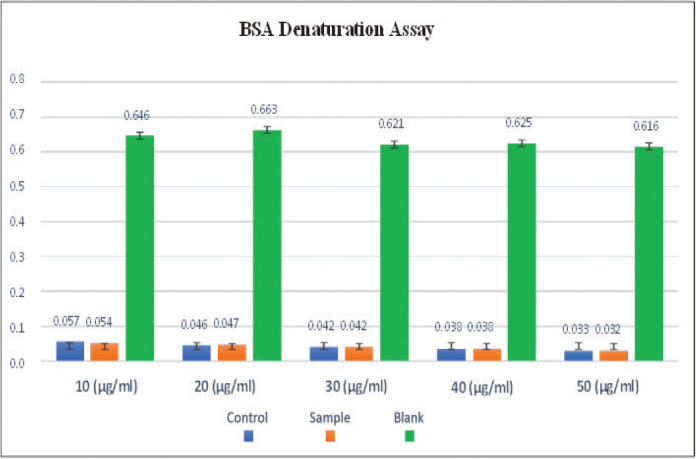
Anti-inflammatory activity (% bovine serum albumin denaturation) of *Amphiroa Fragilisima* at varied concentration. BSA: bovine serum albumin.

The anti-inflammatory response of *A. fragilissima* was tested at five different concentrations (10, 20, 30, 40, and 50 µg/mL) and compared to the blank and control groups (Table 5 and Figure 6). At all concentrations tested, ANOVA revealed significant differences between the blank, control, and *A. fragilissima* groups (*p* < 0.001). However, no significant differences were observed between the control and *A. fragilissima* groups at any concentration (*p* > 0.05). Post hoc analysis showed that the blank group consistently exhibited significantly higher absorbance values than both the control and sample groups across all concentrations (Table 6), indicating lower anti-inflammatory activity in the blank and confirming that both the control and *A. fragilissima* treatments exhibited a comparable and stronger anti-inflammatory effect.

**Table 5:** Anti-inflammatory response for five different concentrations of *Amphiroa fragilissima*.

Concentration (µg/mL)	Group	Mean	Std. deviation	*P*
**@10**	**Control**	0.0565	0.00050	< 0.001
	**Sample**	0.0535	0.00250	
	**Blank**	0.6460	0.01900	
**@20**	**Control**	0.0455	0.00150	< 0.001
	**Sample**	0.0470	0.00100	
	**Blank**	0.6625	0.02350	
**@30**	**Control**	0.0420	0.00100	< 0.001
	**Sample**	0.0415	0.00150	
	**Blank**	0.6205	0.00750	
**@40**	**Control**	0.0380	0.00200	< 0.001
	**Sample**	0.0375	0.00050	
	**Blank**	0.6245	0.01150	
**@50**	**Control**	0.0325	0.00250	< 0.001
	**Sample**	0.0315	0.00050	
	**Blank**	0.6155	0.00150	

**Table 6:** Post hoc analysis on anti-inflammatory responses between three groups at various concentration of *Amphiroa fragilissima*.

Concentra tion (µg/mL)	Group comparing	Group compared	Mean difference	Std. error	*P*
**@10**	**Control**	**Sample**	0.00300	0.00904	0.942
		**Blank**	-0.58950*	0.00904	< 0.001
	**Sample**	**Control**	-0.00300	0.00904	0.942
		**Blank**	-0.59250*	0.00904	< 0.001
	**Blank**	**Control**	0.58950*	0.00904	< 0.001
		**Sample**	0.59250*	0.00904	< 0.001
**@20**	**Control**	**Sample**	-0.00150	0.01111	0.990
		**Blank**	-0.61700*	0.01111	< 0.001
	**Sample**	**Control**	0.00150	0.01111	0.990
		**Blank**	-0.61550*	0.01111	< 0.001
	**Blank**	**Control**	0.61700*	0.01111	< 0.001
		**Sample**	0.61550*	0.01111	< 0.001
**@30**	**Control**	**Sample**	0.00050	0.00364	0.990
		**Blank**	-0.57850*	0.00364	< 0.001
	**Sample**	**Control**	-0.00050	0.00364	0.990
		**Blank**	-0.57900*	0.00364	< 0.001
	**Blank**	**Control**	0.57850*	0.00364	< 0.001
		**Sample**	0.57900*	0.00364	< 0.001
**@40**	**Control**	**Sample**	0.00050	0.00551	0.995
		**Blank**	-0.58650*	0.00551	< 0.001
	**Sample**	**Control**	-0.00050	0.00551	0.995
		**Blank**	-0.58700*	0.00551	< 0.001
	**Blank**	**Control**	0.58650*	0.00551	< 0.001
		**Sample**	0.58700*	0.00551	< 0.001
**@50**	**Control**	**Sample**	0.00100	0.00139	0.763
		**Blank**	-0.58300*	0.00139	< 0.001
	**Sample**	**Control**	-0.00100	0.00139	0.763
		**Blank**	-0.58400*	0.00139	< 0.001
	**Blank**	**Control**	0.58300*	0.00139	< 0.001
		**Sample**	0.58400*	0.00139	< 0.001

## Discussion

Dental caries, periodontal disease, and orthodontic treatment–related complications such as enamel demineralization and soft tissue inflammation remain significant clinical challenges. The search for natural, multifunctional biomaterials that can address microbial, inflammatory, oxidative, and remineralization issues has gained momentum. Marine algae, particularly calcareous red algae, have garnered interest in this context due to their rich phytochemical content and high calcium carbonate composition. *Amphiroa fragilissima*, a calcareous red macroalga, demonstrates promising therapeutic potential owing to its antimicrobial, antibiofilm, antioxidant, and anti-inflammatory properties, as shown in this study. Given its inherent calcium-rich matrix, *A. fragilissima* is particularly relevant for dental and orthodontic applications, where biocompatibility, antimicrobial action, and enamel remineralization are critical [16–18].

The antimicrobial efficacy of *A. fragilissima* extract varied across tested concentrations and bacterial strains. At 25 µg/mL, *E. faecalis* exhibited significantly reduced susceptibility compared to *E. coli*, *S. sonnei*, and *S. mutans*. However, at higher concentrations (50–100 µg/mL), inhibition zones increased uniformly across all strains, suggesting a broad-spectrum bactericidal effect at sufficient dosage. This concentration-dependent activity aligns with previous findings for red algae containing secondary metabolites such as bromophenols, terpenoids, and alkaloids, which are known to disrupt bacterial membranes and interfere with protein synthesis and enzyme activity [19–21].

The antibiofilm activity was also evident and progressively increased with concentration. *A. fragilissima* effectively inhibited biofilm formation by *S. mutans*, *E. coli*, *E. faecalis*, and *S. sonnei*, with maximum inhibition observed at 100 µg/mL. This aligns with the findings of Prithviraj et al., who reported biofilm inhibition by using *Amphiroa* species by targeting bacterial quorum sensing. Their study on *A. fragilissima* demonstrated the potential of marine algal extracts as natural antivirulence agents. Such properties highlight the relevance of *Amphiroa*-based interventions in preventive orthodontic care. The ability to disrupt early biofilm formation is particularly valuable in orthodontics, where fixed appliances can promote plaque retention and white-spot lesions [22]. A qualitative approach was chosen to evaluate biofilm formation because the primary objective of this study was to observe biofilm architecture, coverage, and morphological characteristics, rather than to compare absolute biomass levels.

The antioxidant potential of *A. fragilissima*, assessed via radical scavenging assays, showed a concentration-dependent effect, with activity becoming comparable to the control at higher concentrations (40 and 50 µg/mL). These antioxidant effects are likely due to the presence of phenolic compounds and flavonoids, which help to neutralize reactive oxygen species (ROS) [5, 23]. The algal extract was evaluated against phosphate buffer solution as the negative control. Although its antioxidant activity were comparable to, but not higher than, the positive controls, this does not diminish its potential utility. Its main advantage lies in its ability to modulate biofilm formation on enamel surfaces while maintaining biocompatibility, which is critical for dental applications.

Özay and Pehlivan [24] emphasized that fluctuations in the composition and concentration of secondary metabolites – driven by environmental, physiological, and genetic determinants – substantially influence the biological efficacy of plant and algal extracts. This principle is pertinent to mitigating oxidative insult within inflamed gingival tissues and may consequently support improved longevity of orthodontic adhesive bonds.

The anti-inflammatory effect of *A. fragilissima* was evident, particularly at 50 µg/mL, where both the sample and control showed significantly reduced inflammatory responses compared to the blank group. No significant differences were observed between the sample and control groups across all concentrations, suggesting a consistent anti-inflammatory profile. Previous studies on red algae such as *Halimeda opuntia* and *Halimeda tuna* have demonstrated similar outcomes, with reductions in nitric oxide production and suppression of inflammatory cytokines such as TNF-α in macrophage models [25–27]. These findings suggest that *A. fragilissima* may exert its anti-inflammatory effects by modulating key inflammatory pathways, which could be useful for managing gingival inflammation during orthodontic therapy. In the anti-inflammatory assay, DMSO and aspirin were used as negative and positive controls, respectively. Although the activity of *A. fragilissima* was comparable to the positive control and as mentioned above this does not diminish its potential utility. The main advantage lies in its ability to modulate biofilm formation on enamel surfaces while maintaining safety and biocompatibility as key considerations for dental applications.

A defining characteristic of *A. fragilissima* is its high calcium carbonate content in the form of magnesium-substituted calcite and aragonite. A study of several seaweed species reported that *A. fragilissima* exhibited a calcium concentration of ~11720.4 ppm (i.e. ~1.172% w/w) in dry sample form. In another ecological study the calcified thallus of *A. fragilissima* was described as being deposited in the form of high‑Mg calcite, pointing to significant carbonate (~CaCO₃) content in its skeleton [28]. This feature of high Mg calcite not only supports its structural integrity but also offers potential remineralizing benefits. Calcium and carbonate ions released from algal particulates can aid in enamel repair, especially in demineralized regions adjacent to orthodontic brackets. Prior *in vitro* studies have demonstrated that marine algal powders can promote enamel microhardness recovery and reduce lesion depth, highlighting the potential of such materials as bioactive components in orthodontic adhesives, dental varnishes, and remineralizing toothpastes [18, 29, 30].

Collectively, the results of this study indicate that *A. fragilissima* possesses significant antimicrobial, antibiofilm, antioxidant, and anti-inflammatory properties. Coupled with its calcium-rich matrix, these bioactivities make it a compelling candidate for dental and orthodontic use. It may serve as a bioactive additive in orthodontic adhesives, preventive varnishes, and oral rinses to inhibit bacterial colonization, reduce gingival inflammation, mitigate oxidative stress, and support enamel remineralization. With further *in vivo* validation and formulation development, *A. fragilissima* holds promise as a natural, multifunctional agent in modern dental care and orthodontic therapeutics.

### Limitation

Since this study was an *in vitro* assessment, its *in vivo* application in dentistry cannot be determined. Also, this study did not assess cytotoxicity, long-term stability, or directly compare *A. fragilissima* with standard remineralizing agents.

### Future recommendation

Since all evaluated parameters yielded promising results, further investigation into the remineralizing potential, cytotoxity and long-term stability of this compound is warranted to substantiate its application in dentistry.

## Conclusion

The study underscores the significant therapeutic potential of *A. fragilissima*, exhibiting strong antimicrobial, antibiofilm, antioxidant, and anti-inflammatory activities. Its abundant bioactive compounds, including terpenoids, phenolics, and calcium, contribute to its effective neutralization of free radicals and broad-spectrum antimicrobial effects. These properties highlight *A. fragilissima* as a promising natural agent for oral health applications, with potential benefits in managing biofilm-related infections and inflammatory conditions. Moreover, its calcium-rich composition suggests a valuable role in dental remineralization and orthodontic care. Overall, *A. fragilissima* represents a multifunctional bioactive resource with promising applications in pharmaceuticals, dentistry, and healthcare, meriting further in-depth investigation for clinical and therapeutic use.
